# Phacoemulsification with hydrodelineation and OVD-assisted hydrodissection in posterior polar cataract

**DOI:** 10.1186/s12886-018-0845-8

**Published:** 2018-07-09

**Authors:** Xia Hua, Yongxiao Dong, Jianying Du, Jin Yang, Xiaoyong Yuan

**Affiliations:** 1 Department of Ophthalmology, the Second Hospital of Tianjin Medical University, Tianjin, China; 2 Department of Ophthalmology, the First People’s Hospital of Xianyang, Shanxi, 712000 China; 3 Tianjin Eye Hospital, Tianjin Key Lab of Ophthalmology and Visual Science, Clinical College of Ophthalmology, Tianjin Medical University, Tianjin, 300020 China

**Keywords:** Posterior polar cataract, Posterior capsule rupture, Ophthalmic viscosurgical device, Hydrodissection

## Abstract

**Background:**

To evaluate the results and complications of phacoemulsification with hydrodelineation and ophthalmic viscosurgical device (OVD)-assisted hydrodissection for posterior polar cataract (PPC).

**Methods:**

Medical records of 24 eyes from 17 patients with clinical diagnosis of PPC, who underwent phacoemulsification with hydrodelineation and OVD-assisted hydrodissection, were retrospectively reviewed.

**Results:**

The incidence of posterior capsule rupture (PCR) was 16.67% (4/24): 2 cases occurred during epinucleus removal, and 2 cases occurred during OVD removal after the implantation of the intraocular lens into the bag. No nucleus piece or lens materials dropped into the vitreous during cataract surgery, and no obvious postoperative complications were found during follow-up. All patients had improved best-corrected visual acuity (BCVA) 1 month postoperatively.

**Conclusion:**

OVD-assisted hydrodissection could be an effective technique in phacoemulsification to reduce the incidence of PCR and achieve satisfactory postoperative outcomes.

## Background

Posterior polar cataract (PPC) is a type of developmental cataract characterized by a white, well-defined, distinctive discoid opacity located on or in front of the central posterior capsule (PC) [1]. PPC presents a special challenge to the phaco surgeon due to its high risk of posterior capsule rupture (PCR), vitreous loss, and even nuclear drop during cataract surgery, which can occur because of extreme weakness, pre-existing dehiscence or tight adherence of opacity in the PC [2]. In general, cataract surgery, the incidence of PCR is less than 1% [2], while it is up to 36% in surgery for PPC [3–5].

During phacoemulsification, PCR occurs most often in the removal of the epinucleus [5] or the posterior polar opacity [6] for PPC cases. The recommended strategies [5–8] delay the removal of the posterior polar opacity in the epinuclear plate until complete emulsification of the whole nucleus to minimize the risk for dropping nuclear fragments and losing vitreous. In this study, we retrospectively evaluated the results of our case series of phacoemulsification with hydrodelineation, phacoemulsification of the nucleus, followed by ophthalmic viscosurgical device (OVD)-assisted hydrodissection in PPCs.

## Methods

This retrospective study was approved by the ethics committee of the First People’s Hospital of Xianyang, and all procedures were performed in accordance with the Declaration of Helsinki. From January 1, 2016 to December 12, 2017, patients with a clinical diagnosis of PPC based on slit lamp microscopy who underwent phacoemulsification with hydrodelineation and OVD-assisted hydrodissection were retrospectively reviewed to obtain data on the patients’ demographics, preoperative and postoperative visual acuity, the integrity of the PC, and other complications during surgery.

All surgeries were performed by the same surgeon (YD). After topical anesthesia, a 1-mm side port clear corneal incision was made, followed by injection of about 250 μl of a viscoelastic material Qisheng (Medical Sodium Hyaluronate Gel, Shanghai Qisheng Biological Agent Co., Shanghai, China), a viscous, cohesive gel with 1.5% sodium hyaluronate at a molecular weight of 2,000,000 to 2,500,000 Da, providing approximately 450,000 mPa·s at a shear rate of 0.01 Hz at 25 °C into the anterior chamber. A 2.8-mm, 3-stepped, clear corneal incision was made 90 degree to the right of the side port incision. Capsulorhexis was started by pinching the anterior capsule by the forceps, and continued with a 5.0-mm continuous curvilinear capsulorhexis (CCC), taking special care to avoiding viscoelastic escape from the incision. Only hydrodelineation was performed to separate the epinucleus and nucleus. A venture system phaco machine (Stellaris, Bausch & Lomb, Rochester, New York, USA) was set to a perimeter lower than normal at power 35%, vacuum 280 mmHg, and bottle height 70 cm. The phaco-chop technique was used. After the first division of the nucleus, we rotated the phaco tip towards one-half of the nuclear piece, followed by chopping and emulsifying in situ, avoiding any rotation of the lens pieces. Then for the residual nucleus pieces, we left the integrated posterior epinucleus in situ. The above viscoelastic was injected carefully between PPC and PC from 3 o’clock to 9 o’clock, to lift the epinucleus with the posterior opaque and push down the PC. For large-sized PPC, bi-directional OVD-assisted hydrodissection was performed to release the synechia between PPC and PC. If the PC was judged to have integrated tentatively, routine irrigation and aspiration of the posterior epinucleus and cortex was performed by lowering the vacuum at 280 mmHg. A foldable IOL was implanted into the capsular bag.

**Fig. 1:**
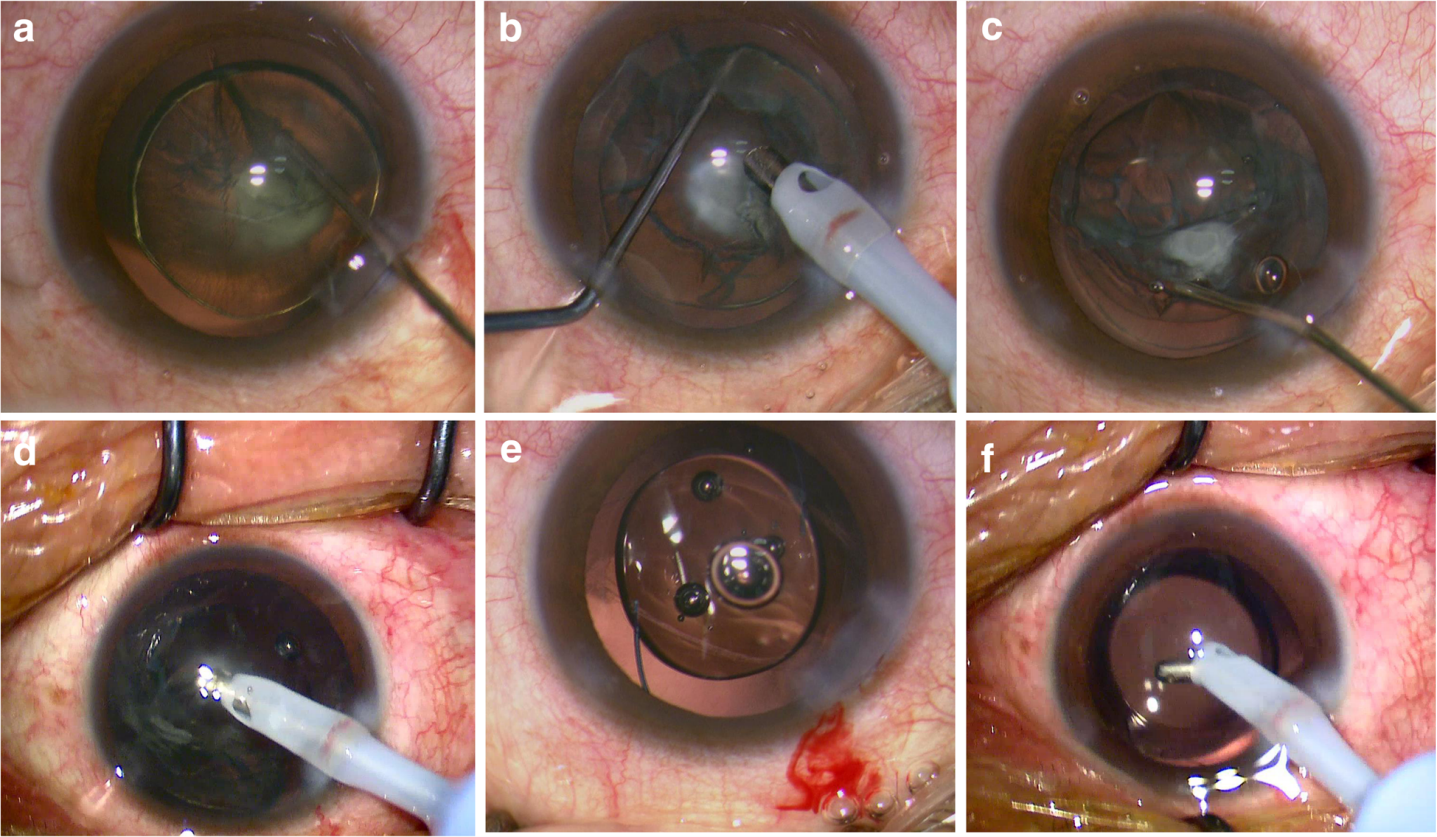
**a** A golden ring followed hydrodelineation to separate the epinucleus and nucleus. **b** Phacoemulsification of the nucleus with the phaco-chop technique, leaving the posterior capsular cataract (PPC) with the epinucleus in situ. **c** Ophthalmic viscosurgical device (OVD)-assisted hydrodissection. The PPC was lifted with the epinucleus by injecting viscoelastic material between the PPC and the posterior capsule. **d** Irrigation/aspiration of the lifted PPC with the epinucleus. **e** A 3-piece acrylic IOL implantation into a sulcus with posterior capsular rupture. **f** A 1-piece acrylic IOL into a capsular bag with an intact posterior capsule

If PCR was found during surgery, a dispersive viscoelastic (Viscoat, Alcon, Fort Worth, Texas, USA) was injected beside the OVD to see if the tear could be converted to a continuous curvilinear capsulorhexis. Vitrectomy with a high cutting rate of 800 cpm, low vacuum of 100 mmHg, and bottle height of 50 cm was then performed until the anterior chamber was free of vitreous, if necessary with viscoelastic instead of fluid irrigation, follow by IOL in the sulcus (Fig. 1).

After surgery, all the patients were given TobraDex (Alcon, Fort Worth, Texas, USA) qid. For the first week, which was tapered over the following 4 weeks. All the patients were routinely examined on the first day, first week, first month, and 6 months postoperatively. The data from the first month were evaluated here. And the comparisons of BCVA (logMAR) between 1 month postoperation and pre-operation were analyzed with Student’s t-test. *P* value less than 0.05 (two tails) was considered as statistical difference.

## Results

In total, 24 eyes in 17 patients with clinically diagnosed PPC were enrolled in this study (Table 1).

**Table 1:** shows the clinical data of these patients

Demographics	
Age (years, mean ± SD)	62.12 ± 13.28
Male/female (n)	10/7
Right/left (n)	14/10
PCR	4 (16.67%)

According to the Emery-Little classification, the nuclear sclerosis (NS) grades were II in 14 eyes, III in seven eyes, and IV in three eyes.

In all patients, CCC and hydrodelineation were performed uneventfully. The nucleus was successfully emulsified and aspirated in all cases of PPC. No PCR was noticed, and no vitreous prolapse or loss was found in this stage or before. In 22 eyes, OVD-assisted hydrodissection was successfully performed, as the OVD was injected between the capsular rim and the epinucleus. The posterior opacity was floated up and curled towards the main incision. The PC was pushed down, and a gap generated by the OVD suggested the PC was integrated at this stage. The I/A hand piece was inserted, and the epinucleus and cortex were removed by the phaco program.

In two cases, PCR was found during the OVD-assisted hydrodissection, and dense synechia was noticed between the PPC and PC. The OVD was carefully injected, and the PC was not pushed down; instead, a horizontal or an oblique rupture was noticed. A little more OVD was injected upon the PCR, followed by manual I/A of the epinucleus and residual cortex by a Simeco cannula. Of these two cases, the vitreous prolapsed in one case, and anterior vitrectomy was needed. In the other case, PPC was successfully floated up, and when automatic I/A was being performed, PCR occurred, followed by more dispersive OVD injection and manual I/A. A one-piece acrylic IOL was implanted into the bag if the PC was intact, and a three-piece acrylic IOL with PMMA haptics was inserted into the sulcus if PCR occurred. Then, OVD in the anterior and posterior chambers was removed by the I/A hand piece or Simeco canula.

After removing the OVD and before reforming the AC, two cases with acrylic IOL in the capsular bag were noticed with PCR. Therefore, some dispersive OVD was injected upon the PCR, and the one-piece IOL was changed to the three-piece one in the sulcus.

In all patients, BCVA (logMAR) 1 month postoperation (0.15 ± 0.12) was much improved compared with pre-operation (0.58 ± 0.23) (*t* = 8.23, *p* < 0.0001). In addition, no obvious posterior segment complication was found, even in eyes with PCR. In 7 cases, minimal corneal edema and anterior chamber flare were seen on day 1 postoperation, but they had disappeared by 1 week postoperation. Temporary high IOP was found in 7 eyes, 2 of which had PCR. It was controlled by tapping some of the aqueous, and it recovered in 2 days. In 2 eyes with uneventful surgery, obvious macular degeneration was found after surgery. Both had a BCVA of 20/50, with deformation by 1 month postoperation.

## Discussion

Because of the extremely thin or even defective local posterior capsule of PPC, PCR occurs at a high rate during ECCE, phaco surgery, and even femtosecond laser-assisted cataract surgery [3, 6, 9, 10]. PCR is inevitable for some cases of PPCs due to pre-existing posterior capsule defects or strong synechia between the posterior opacity and posterior capsule. Special care should be taken at all steps of cataract surgery for such cases.

In our study, all procedures started with a side port incision followed by injecting viscoelastic into the anterior chamber. We believe that the possibility of anterior chamber collapse might be lower with this approach than starting from the main incision, so this approach might be helpful for avoiding the rupture of a weak PC. Overloading viscoelastic injection should be avoided. For capsulorhexis, we preferred to use a forceps with a sharp tip, starting by pinching the anterior capsule instead of applying downward force. During the whole maneuver, it was important not to exert pressure on the incision and the operated eye; otherwise, the viscoelastic might overflow and the anterior chamber prolapse. a 5–5.5-mm capsulorhexis was performed. In our opinion, a too-large capsulorhexis is not suggested; although it can cause minimal turbulence in the capsular bag during phacoemulsification, if PCR occurs, not enough anterior capsule rim will exist to support the IOL in the sulcus [11]. Most previous studies suggested a 5.0- to 5.5-mm circular curvilinear capsulorhexis. Vasavada preferred 4.5 mm [5]. Pong and Lai suggested a 5.5- to 6.0-mm CCC for a hard nucleus [12]. Singh described an oval capsulorhexis technique with all grades of nuclear sclerosis for PPC with preexisting PCR, with good results [11]. In our cases, it was not difficult to perform the hydro process and phaco chop with a 5.5-mm-diameter anterior capsule opening, even for a moderate-hardness nucleus. Routine hydrodissection may cause a sudden fluid wave in the weak or defective PC of such cases, which should be avoided right after capsulorhexis. Many surgeons [7, 9, 13, 14] suggest only hydrodelineation at this stage for PPC. We performed conventional hydrodelineation with the cannula penetrating into the lens. As the fluid was injected from outside to inside, usually a golden ring was noticed, which indicated a successful hydrodelineation. A layer of epinucleus was in front of the PC, and the posterior polar opacity was left in situ. Then, phacoemulsification with lower vacuum and lower bottle height was started.

We selected the phaco chop technique in this case series. As the nucleus was divided into pieces, the nuclear material was emulsified in situ, without rotating with the epinuclear plane in front of PC, all of which might exert minimal stress on the capsular bag, especially the weak PC. During the process, it was important not to exert stress on the capsular bag and not to rotate the lens substance until all the nucleus was emulsified and aspirated. Another important issue was to keep the anterior chamber stable. Some viscoelastic could be injected through the side port before withdrawing the phaco tip.

Epinuclear removal might be the most dangerous and difficult part of PPC surgeries [15]. Some surgeons use a phaco tip with very low aspiration flow rate, vacuum, ultrasound power, and bottle height, to strip the epinucleus from the PC [5, 8]. In our case series, a medical sodium hyaluronate gel, Qisheng, was used as the fluid for hydrodissection at 180 degrees opposite the main incision to alleviate the epinucleus with posterior opacity from the PC. As more OVD was injected, the PC was noticed to be pushed down and the posterior opacity with the epinucleus flowed up, which was followed by the I/A tip instead of the phaco tip to remove the epinucleus gradually, and the residual cortex as well.

Posterior capsule polishing should be avoided, even the PC is intact with some opacity, because of the weak nature of such PCs [5, 6, 13]. Most PCRs in our cases were noticed during this stage, and some Viscoat was injected between the PCR plane and epinucleus. The epinucleus was then removed by manual dry aspiration with the Simcoe cannula if no or minimal vitreous was left out. Otherwise, anterior vitrectomy with a high cutting rate and low vacuum and bottle height was performed until the anterior chamber was free of vitreous. One piece of acrylic IOL was then inserted into the bag for the intact PC, or a three-piece of IOL (acrylic optical IOL with PMMA haptics) was inserted into the sulcus.

Different surgical techniques have been recommended to prevent or reduce the incidence of PCR in PPC cases. Osher et al. reported a 26% incidence of PCR during cataract surgeries with a slow motion phacoemulsification, combined with lower settings of aspiration, vacuum, and infusion pressure [6]. Using a lambda technique with dry aspiration, Lee and Lee’s case series showed a PCR ratio with 11.1% [14] . And Vasavada’s group favored inside-out delineation with culmulative surgical experience, they reduced the incidence of PCR to 8% [16]. Compared with other reports of different surgical techniques for PPC, the PCR ratio in our case series is 16.67%, among which, 8.34% was found before IOL implantation, and the other 8.34% occurred after the removal of OVD in the anterior chamber.

We observed two cases of PCR when the anterior chamber collapsed after removing all the OVD. In both cases, the PC was intact after the one-piece acrylic IOL was inserted into the bag, and then, routine I/A of viscoelastic material was performed normally. After withdrawal of the I/A tip, the anterior chamber was shallowed, and then PCR was found. We believe that this kind of PC is fragile by nature and cannot endure pressure from the posterior segment and IOL as the anterior chamber disappears [9].

Only one eye did not reach satisfactory postoperative visual acuity, due to macular degeneration with uneventful cataract surgery. All other eyes had improved BCVA, whether PCR existed or not. With our technique of hydrodelineation first, then phacoemulsification of the nucleus, followed by OVD-assisted hydrodissection, the epinucleus with posterior opacity was easy to remove, and it was safe to handle the lens materials, even those with pre-existing PCR or PCR that occurred during this stage, since the chance of dropping the nucleus was minimal and leakage of vitreous was effectively pushed back.

## Conclusion

In conclusion, PCR is sometimes inevitable in cases of PPC. We adopted different techniques to minimize the damages to the affected eyes. The goals of the surgery are to remove the PPC safely and to keep the integrity of the PC or reduce the chance of dropping lens materials and the loss of vitreous. Phacoemulsification with hydrodelineation and OVD hydrodissection for removal of the epinucleus was an effective treatment for PPCs.

## Abbreviations

BCVA: Best-corrected visual acuity
CCC: Continuous curvilinear capsulorhexis
OVD: Ophthalmic viscosurgical device
PC: Posterior capsule
PCR: Posterior capsule rupture
PPC: Posterior polar cataract
